# The health effect of relaxing entry regulation for private hospitals and its nonlinear characteristics: evidence from China

**DOI:** 10.3389/fpubh.2025.1432806

**Published:** 2025-01-15

**Authors:** Wanwen Jia

**Affiliations:** ^1^China Institute of Regulation Research, Zhejiang University of Finance and Economics, Hangzhou, China; ^2^The New Type Key Think Tank of Zhejiang Province “China Research Institute of Regulation and Public Policy”, Zhejiang University of Finance and Economics, Hangzhou, China

**Keywords:** entry regulation, private hospital, health, hospital competition, threshold test

## Abstract

**Introduction:**

Relaxing entry regulation for private hospitals and fostering competition in the healthcare market are crucial prerequisites for addressing the diverse healthcare demands of the population and promoting the development of a Healthy China. This study aims to comprehensively evaluate the health effect of relaxing entry regulation for private hospitals and to examine its nonlinear characteristics.

**Methods:**

Using panel data from 31 provinces in mainland China, this study employs a fixed effects panel data model to investigate the health effect of relaxing entry regulation for private hospitals. To examine the potential nonlinear relationship, a panel threshold model is also employed. This study also employs a dynamic panel model as the econometric method to perform regression analysis for robustness testing.

**Results:**

The empirical findings indicate that relaxing entry regulation for private hospitals significantly improves the residents’ health level. This conclusion remains robust when different explanatory variables, measurement models and modified samples are applied. The heterogeneity analysis reveals that the health benefits of private hospital market entry are more pronounced in Central-Western region, regions with high medical resource endowment, and regions with High-quality competition. Furthermore, the health effect of relaxing entry regulation for private hospitals exhibits nonlinear characteristics: it weakens as economic development level reaches a certain threshold and becomes more pronounced as educational attainment reaches a specific threshold.

**Discussion:**

Based on these empirical results, Chinese provinces can be classified into three distinct types to guide the development of differentiated policies tailored to local conditions. This study provides valuable theoretical insights into promoting a fair competition mechanism in the healthcare market, enhancing the quality of private hospitals, and improving the overall health level of residents in China and other developing countries facing similar challenges.

## 1 Introduction

Due to information asymmetry between healthcare providers and patients as well as, externalities and uncertainty, China’s healthcare market has long been subject to government regulation (1, 2), covering various aspects, including quality, entry, and pricing (3). Among these, entry regulation plays a crucial role in ensuring the diversification of healthcare supply and in fostering a fair and orderly competitive market environment (4). China classifies its hospitals into public and private categories based on their economic models. In contrast to a liberalized market economy, both public and private hospitals in China remain subject to government regulation. The government plays a central role in guiding the provision of healthcare services through policy interventions, while also exerting significant influence over the market entry of private hospitals, the allocation of medical resources, the pricing of healthcare services, and the overall competition within the healthcare market. In recent years, to increase the supply and improve the efficiency of healthcare services, the Chinese government has progressively relaxed entry regulation for private hospitals, encouraged and guided social capital to establish medical institutions, and fostered competition within the healthcare market.

In 2010, the General Office of the State Council of China forwarded the “Opinions on Further Encouraging and Guiding Social Capital to Organize Medical Institutions,” emphasizing the need to remove policy barriers hindering the development of private hospitals, thereby ensuring that private hospitals receive the same treatment as public hospitals in terms of market entry, operational practice and development. In 2015, the General Office of the State Council of China issued the “Several Policies and Measures to Promote the Accelerated Development of Socially-Run Medical Institutions,” noting that the development of socially-run medical institutions is still far from achieving the goal of establishing a diversified medical institution landscape, and that systematic obstacles and policy constraints persist. Consequently, in 2017, the General Office of the State Council of China again issued the “Opinions on Supporting Social Forces to Provide Multi-level and Diversified Medical Services,” proposing the relaxation of market entry regulation for private hospitals. In 2024, the State Council of China released the “Key Tasks for Deepening the Pharmaceutical and Healthcare System Reform in 2024,” outlining policies to promote and regulate the development of private hospitals.

**Figure 1:**
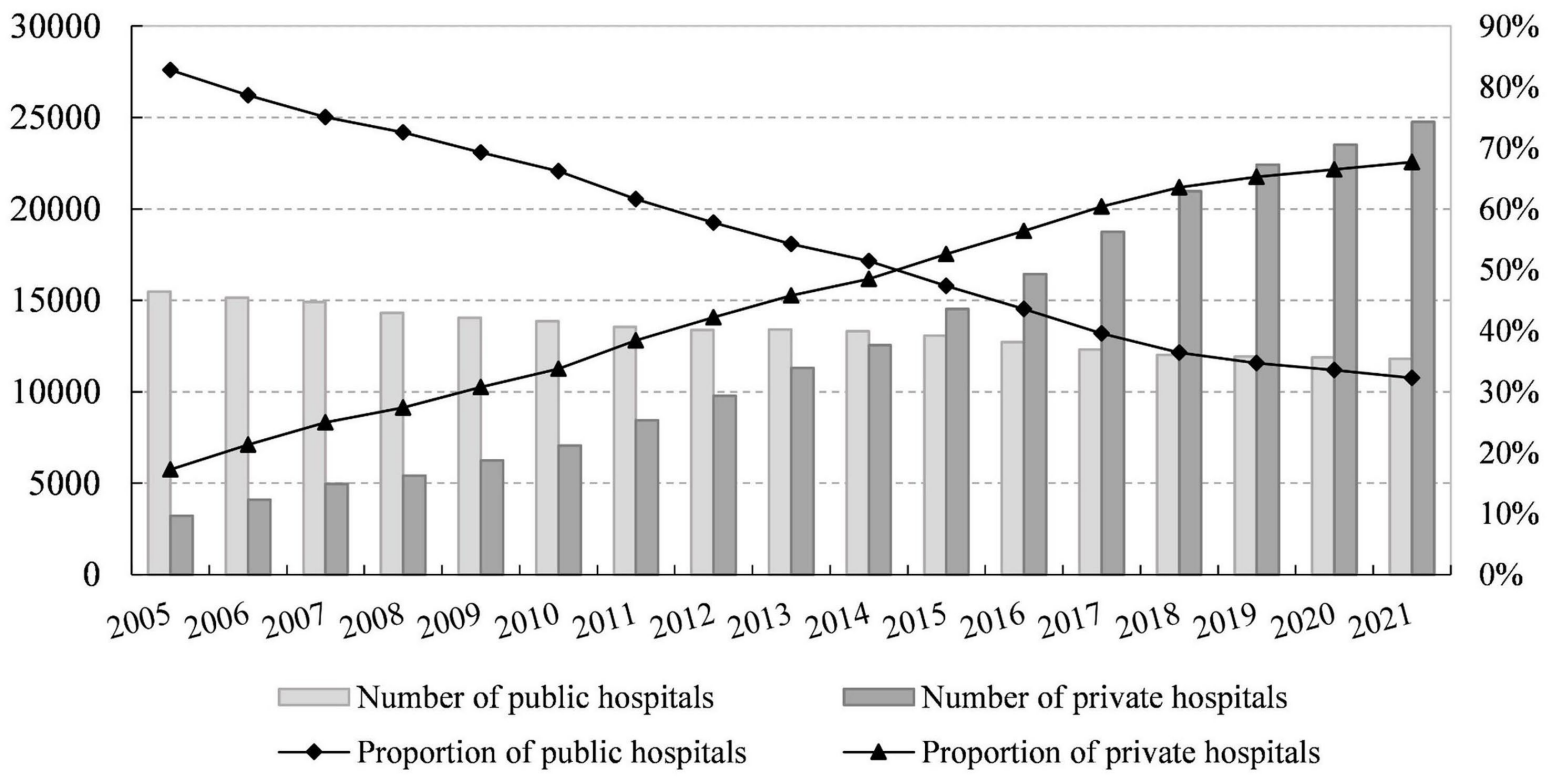
Trends in the number and proportion of public and private hospitals in China, 2005-2021. Data Source: China Healthcare Statistical Yearbook.

Against the backdrop of relaxed entry regulation for private hospitals, the number of private hospitals has increased significantly, as shown in Figure 1, which illustrates the trend in the number of institutions and the proportion of public and private hospitals in China from 2005 to 2021.

Figure 1 demonstrates gradual decrease in the proportion of public hospitals, from 82.78% in 2005 to 32.28% in 2021, while the proportion of private hospitals has steadily increased from 17.22% in 2005 to 67.72% in 2021. Since 2015, the number of private hospitals in China has surpassed that of public hospitals, with a continuous annual increase. In recent years, the Chinese government’s policies to relax entry regulation for private hospitals and encourage social capital in healthcare services have yielded notable results.

The “Healthy China 2030 Plan” issued in 2016 explicitly stated that advancing the construction of a Healthy China is a crucial foundation for building a moderately prosperous society and comprehensively achieving socialist modernization. In 2019, the State Council of China issued the “Opinions on the Implementation of Healthy China Actions,” which underscored the strategic importance of building a Healthy China. The report of the 20th Party Congress reiterated that public health is a critical symbol of national prosperity and strength, and that safeguarding public health should be placed as a priority in national strategic development. Improving residents’ health remains the ultimate goal of healthcare development. In this context, how to stimulate competition in the healthcare market and meet the diverse medical demands of the population are pressing issues that must be addressed in realizing the strategic goal of a Healthy China. These are also critical topics for academic focus. Women’s health indicators are internationally recognized health metrics. According to the Sixth National Health Service Survey Report of China, the hospital delivery rate among pregnant women has significantly increased, rising from 38.7% in 1993 to 98.6% in 2018. Meanwhile, the proportion of deliveries taking place in private hospitals has also increased. This indicates that the health protection level for pregnant women in China has improved in recent years and suggests a potential correlation between the development of private hospitals and improvements in health levels.

Accordingly, this study utilizes panel data from China to perform both theoretical and empirical analyses, seeking to address the following questions: What is the present impact of relaxing entry regulation for private hospitals on health level? Are there heterogeneous effects? Does this impact exhibit nonlinear characteristics?

The remaining structure of this study is organized as follows. Section 2 reviews the relevant literature and formulates research hypotheses. Section 3 outlines the research methodology. Section 4 presents and discusses the empirical results. Section 5 examines the nonlinear characteristics of the relationship. Section 6 provides the conclusions, policy implications, limitations, and recommendations for future research.

## 2 Literature review and research hypotheses

### 2.1 Literature review

The trend of reform, characterized by the relaxation of entry regulation for private hospitals, has progressively garnered the attention of the academic community. Existing studies have examined the competitive effects of private hospital market entry, focusing primarily on its impact on healthcare costs, quality, and efficiency. Given the unique characteristics of the healthcare market, the competitive effects between private and public hospitals differ from those observed in other markets (5).

Several empirical studies have demonstrated that workload indicators in for-profit hospitals significantly reduce per capita healthcare costs (6), and that privatization fosters price competition, thereby reducing patients’ hospitalization and out-of-pocket expenses (7). However, other studies have also pointed out that the healthcare services market possesses unique characteristics: patients are not price-sensitive, and competition is primarily centered on quality (8–10). Moreover, service quality constitutes a crucial strategic factor in the competition among healthcare institutions (11). As hospitals engage in quality competition, they increase their investment various healthcare resources to attract patients. Healthcare institutions are incentivized to recover investment costs by expanding the volume of healthcare services (12), which consequently leads to increased healthcare costs. Moreover, it is argued that the impact of competition between private and public hospitals on healthcare costs remains inconclusive. Competition in the healthcare services market reduces individual healthcare costs and government health expenditures, but has an insignificant impact on social expenditures, while simultaneously increases the country’s total health costs (13). In a market characterized by segregated competitive structures, an increase in the number of firms does not necessarily result in lower market prices. Therefore, merely increasing the number of hospitals may not effectively reduce healthcare service prices (14).

It is widely acknowledged that relaxing entry regulation for private hospitals and promoting market competition between public and private hospitals contribute to improving the quality of healthcare services (15–17). Jiang (18) demonstrated through empirical analysis that the competition between for-profit and non-profit hospitals not only reduced the mortality rate of admitted patients but also increased the cure rate, thereby improving the quality of inpatient services. However, some scholars argue otherwise. Dranove and Satterthwaite (19) argued that although quality competition in healthcare institutions introduces advanced medical technology, doctors’ over-reliance on equipment may reduce their motivation to learn, potentially diminishing the quality of healthcare. Additionally, Brekke et al. (20) contended that competition does not improve the quality of healthcare when hospitals’ marginal costs are incremental. The entry of private hospitals into the market not only intensifies competition but also promotes market fairness, positively influencing the efficiency of healthcare services (1, 5) and significantly enhancing patient welfare (17). Existing literature examines the competitive effects of private hospitals entering the market across several dimensions. However, as noted by Wang and Wei (12), the specificity of healthcare services and patient preferences significantly influence these effects, and no consistent conclusions exist regarding the outcomes of competition between public and private hospitals.

Integrating health into all policies and establishing the concept of “great health” are pressing demands of contemporary society (21). Research on health primarily focuses on its determinants, with existing literature mainly examining on financial health expenditures (22, 23), medical insurance (24), educational attainment (25), income levels (26, 27), international trade (28), and other factors. However, few studies have addressed health issues from the perspective of government regulation. In terms of the impact of private hospitals entering the market on health, Guo and Wu (7) concluded that neither the number of private hospitals nor their market share significantly affect residents’ health or satisfaction with medical care.

The aforementioned studies have extensively explored and discussed the effects of competition between public and private hospitals, however, there remains considerable room for further exploration. First, a review of the existing literature indicates that empirical studies on hospital competition have predominantly focused on the impacts on medical costs and quality, however, analyses of health effects remain relatively underdeveloped. Second, the evaluation of the effects of relaxing entry regulation for private hospitals is lacking systematic research grounded in market competition theory and integrated with a health perspective, which limits the provision of scientific basis for policy optimization. Third, the health effects of relaxing entry regulation for private hospitals are complex and multifaceted. Therefore, further investigation is required to achieve a more comprehensive understanding of the heterogeneous and nonlinear characteristics of these effects.

Building on the identified research gaps, this study adopts government regulation as a starting point and uses empirical data from China to systematically analyze and evaluate the health effect of relaxing entry regulation for private hospitals. The findings of the study will provide an important reference for enhancing the diversity of medical service supply and establishing a reasonable market competition framework in China.

The marginal contributions of this study are primarily in three aspects. (1) This study systematically evaluates the health effects of relaxing entry regulation for private hospitals, comprehensively examines their heterogeneous effects from multiple perspectives, and provides important empirical support for policy enhancement. (2) Using the panel threshold model, this study reveals the nonlinear characteristics of the health effects of relaxing entry regulation for private hospitals. By analyzing the relationship between economic development level, educational attainment, and threshold values, this study categorizes Chinese provinces into three typical regional types. This classification provides a theoretical foundation for formulating and optimizing differentiated regulatory policies for various regions and designing more targeted development policies for private hospitals tailored to local conditions, thereby effectively stimulating the vitality of the healthcare service market. (3) This study investigates the issue of competition within the healthcare services market from the perspective of relaxing entry regulation for private hospitals. It not only enriches the application of government regulation theory in the field of healthcare but also expands the research perspective on health-related issues.

### 2.2 Research hypotheses

Based on the analysis of the policy context and the literature review, this study examines the relationships among the government, hospitals, and patients within the framework of relaxing the entry regulation for private hospitals. For the government, which plays a guiding role in the healthcare market, relaxing entry regulation for private hospitals leads to an increase in the number of private hospitals. This, in turn, results in an increase in both the quantity and diversity of medical service supply, thereby generating a direct demand-release effect. Competition in the hospital market has significantly intensified for both public and private hospitals. In response, hospitals are incentivized to improve the quality and efficiency of medical services, thereby creating an indirect market competition effect. For patients, the enhanced accessibility to medical services is expected to impact their health outcomes. Given the variations in economic development level and educational attainment across different regions of China, the health effects of relaxing entry regulation for private hospitals may exhibit threshold characteristics. Based on these observations, this study proposes the following two research hypotheses:

*H1*: With the relaxation of entry regulation for private hospitals, intense hospital competition has a positive effect on the health levels of residents.

*H2*: The health effects of relaxing entry regulation for private hospitals exhibit certain nonlinear characteristics.

## 3 Methods

### 3.1 Model setting

#### 3.1.1 Fixed effects model

The results of the modified Hausman test and the Wald statistic test based on over-identification indicate that the data analyzed in this study are more appropriate for a fixed effects model than for a random effects model. Consequently, this study primarily employs the fixed effects model of the static panel for parameter estimation. The econometric model is specified in Equation 1:


(1) $$\mathrm{Healt}h_{\mathrm{it}}=\partial_{0}+\partial_{1}Comp_{\mathrm{it}}+\partial_{c}\mathrm{Contro}l_{\mathrm{it}}+\mu_{i}+\lambda_{t}+\varepsilon_{\mathrm{it}}$$

Where $\mathrm{Healt}h_{\mathrm{it}}$ denotes the health level of province i in period t . $Comp_{\mathrm{it}}$ stands the degree of relaxing entry regulation for private hospitals of province i in period t . $\mathrm{Contro}l_{\mathrm{it}}$ is a set of control variables: population aging ( aging ), population density ( den ), educational attainment ( edu ), health insurance coverage rate (*insur*), Per capita GDP ( pgdp ), and the proportion of tertiary hospitals ( terh ). $\mu_{i}$ represents province fixed effects, $\lambda_{t}$ represents time fixed effects, and $\varepsilon_{\mathrm{it}}$ denotes the random error term.

#### 3.1.2 Panel threshold model

To examine the potential nonlinear relationship between relaxing entry regulation for private hospitals and health level, this study employs the panel threshold model introduced by Hansen (29). This model addresses the structural breaks in the economy by incorporating a threshold value as an unknown variable in the regression equation, thereby creating a segmented function. This model is particularly useful for investigating whether the impact of relaxing entry regulation for private hospitals on health level is influenced by various factors. The specification of the panel threshold model is given in Equation 2:


(2) $$\begin{array}{l} \mathrm{Healt}h_{\mathrm{it}}=\alpha_{0}+\alpha_{1}Comp_{it}\cdot I\left(C_{\mathrm{it}}\leq \gamma \right)+\alpha_{2}Comp_{\mathrm{it}}\cdot I\left(C_{\mathrm{it}}>\gamma \right)\\ \;+\alpha_{c}\mathrm{Contro}l_{\mathrm{it}}+\mu_{i}+\varepsilon_{\mathrm{it}} \end{array}$$

Where $C_{\mathrm{it}}$ is the threshold variable of province i in period t , γ is the threshold value to be estimated, and $I\left(-\right)$ is the indicator function, where I takes 1 when the condition in parentheses is satisfied, and 0 otherwise. The rest of the variables have the same meaning as in Equation 1.

### 3.2 Variable selection and data description

#### 3.2.1 Variable selection

##### 3.2.1.1 Explained variable: health level (*Health*)

Health level is a crucial indicator of a country’s economic and social development. Therefore, its contribution to improving the population’s health level should be the ultimate criterion for evaluating the healthcare system (30). Health is fundamental to individuals’ overall development and serves as a primary resource for society (31). The National Health Service Survey Report of China emphasizes that the health of women is essential for sustainable human development. Infant and child mortality rates, along with maternal mortality rates, are internationally recognized as core health indicators. Referring to the studies of Zheng and Shen (23), Chen and Jin (32), and considering the availability of data, this study uses the natural logarithm of maternal mortality rate as a measure of the population’s health level. This choice is based on the fact that such a population is more sensitive to policy changes and can more effectively capture the impact of relaxing entry regulation for private hospital on the health level of residents.

##### 3.2.1.2 Core explanatory variable: hospital competition (*Comp*)

This study uses the degree of hospital competition as a proxy variable to represent the relaxation of entry regulation for private hospitals. Drawing on existing studies (12, 13, 33), this study utilizes the Herfindahl–Hirschman Index (HHI) to quantify the degree of competition between public and private hospitals. The HHI is a widely used measure of market concentration, where a higher value indicates greater market concentration and reduced competition. This study primarily focuses on the competitive effect between private and public hospitals under the framework of relaxed entry regulation for private hospitals in China, utilizing the number of public and private hospitals to represent their respective market shares. The formula for calculating the HHI, a metric of market concentration, is presented in Equation 3:


(3) $$HHI={\left(\frac{\mathrm{PUB}}{\mathrm{PUB}+PRI}\right)}^{2}+{\left(\frac{PRI}{\mathrm{PUB}+PRI}\right)}^{2}$$

Where PUB denotes the number of public hospitals, while PRI represents the number of private hospitals. Hospital competition is expressed as Comp = 1-HHI, which quantifies the degree of competition between public and private hospitals based on the number of healthcare institutions.

##### 3.2.1.3 Control variables

Factors such as economic conditions, social capital, medical service utilization, and health resource allocation collectively influence residents’ health levels (34). Grounded in human capital theory, Grossman (35) constructed the health demand model, which provides a theoretical framework for understanding the relationships between age, education, income, and health. Gertler (36) and Mwabu (37) extended Grossman’s health demand theory into empirical analyses. Subsequent studies further incorporated income, demographic characteristics, and latent demand into explanatory frameworks (38–40). Building on the above research, this study identifies the following variables as control variables, as detailed in Table 1.

**Table 1:** Variable definitions.

Variable type		Variable	Variable description	Definition
Dependent variable		Health	Health level	The natural logarithm of maternal mortality rate
Independent variable		Comp	Relaxing entry regulation for private hospitals	Degree of hospital competition between public and private hospitals: 1-HHI
Control variable	Demographic characteristics	aging	Population aging	Individuals aged 65 and above / total population
		den	Population density	The natural logarithm of the number of permanent residents per square kilometer at the end of the year
		edu	Educational attainment	Population aged 6 and above with a junior college degree or higher / total population
	Financial support	insur	Health insurance coverage rate	Number of urban workers with basic health insurance / total population
	Economic development level	pgdp	Per capita gross domestic product	The natural logarithm of the region’s per capita GDP
	Supply factor	terh	Development of tertiary hospitals	Number of tertiary hospitals / total number of hospitals

#### 3.2.2 Data source

The research data in this study are primarily derived from the China Healthcare Statistical Yearbook, China Healthcare Development Statistical Bulletin, China Statistical Yearbook, China Population & Employment Statistical Yearbook, and China Labor Statistical Yearbook. Panel data can partially mitigate multicollinearity among variables. This study employes a dataset consisting of panel data from 31 provinces in mainland China (excluding Hong Kong, Macau, and Taiwan) over the period from 2010 to 2019. The selection of this specific timeframe was informed by two primary considerations. First, it was due to data availability. Specifically, some key indicators, such as the number of private hospitals, only became publicly accessible data starting in 2010. Second, it aimed to ensure the stability and reliability of the research findings. Notably, the outbreak of COVID-19 in China significantly impacted certain indicators, resulting in considerable variability in the data. Consequently, data collection for this study was limited to the end of 2019 to minimize potential confounding effects arising from the extraordinary circumstances of the public health emergency.

Logarithmic transformations are applied to certain variables to mitigate the effect of heteroskedasticity. To account for inflation, this study adjusts per capita GDP to the 2010 base year using the regional per capita GDP index. The descriptive statistics of the variables are summarized in Table 2.

**Table 2:** Descriptive statistics of variables.

Variables	Mean	Min	Max	SD	Sample size
Health	2.591	0.095	5.197	0.734	310
Comp	0.448	0.076	0.500	0.070	310
aging	0.099	0.048	0.163	0.023	310
den	8.982	7.180	10.213	0.645	310
edu	0.132	0.024	0.505	0.072	310
insur	0.217	0.078	0.781	0.135	310
pgdp	10.642	9.464	11.905	0.476	310
terh	0.077	0.019	0.158	0.031	310

As illustrated in Table 2, the explained variable, health level, ranges from a minimum of 0.095 to a maximum of 5.197, indicating substantial disparities in health levels across various provinces in China over the observed years. The explanatory variable, hospital competition, varies from 0.076 to 0.500, reflecting notable variation in the development of private hospitals across provinces in China. Furthermore, considerable inter-provincial disparities are observed in population aging, health insurance coverage, economic development level, and the proportion of tertiary hospitals. These summary statistics provide a comprehensive overview of the dataset and establish a foundation for subsequent empirical analysis.

## 4 Empirical results

### 4.1 Baseline regression results

Utilizing a fixed effects model, this study empirically examines the causal relationship between relaxing entry regulation for private hospitals and health level, with the findings presented in Table 3. Column (1) of Table 3 demonstrates the effect of relaxing entry regulation for private hospitals on health level without including of control variables, while columns (2) to (4) of Table 3 progressively incorporate control variables, time-fixed effects, and province-fixed effects, respectively. The results remain consistent when considering various fixed effects and control variables. Column (4) of Table 3 indicates that hospital competition significantly improves residents’ health level within the context of relaxed entry regulation for private hospitals in China. Thus, Hypothesis 1 is confirmed.

**Table 3:** Results of baseline regression.

Variables	(1)	(2)	(3)	(4)
Comp	−1.911^***^ (0.517)	−1.110^*^ (0.597)	−1.066^**^ (0.535)	−0.930^**^ (0.452)
aging		0.825 (2.543)	−2.194 (2.665)	4.108 (2.760)
den		−0.073 (0.076)	−0.124^*^ (0.070)	−0.017 (0.054)
edu		−0.208 (0.958)	−1.518 (1.321)	−3.187^*^ (1.707)
insur		−0.871 (0.664)	1.083 (1.290)	0.464 (2.197)
pgdp		−0.606^***^ (0.146)	−1.304^***^ (0.295)	−1.295^***^ (0.431)
terh		−0.692 (1.681)	−1.102 (1.630)	1.811 (1.732)
Cons	3.448^***^ (0.250)	10.384^***^ (1.442)	18.064^***^ (3.133)	16.526^***^ (4.818)
control variables	NO	YES	YES	YES
time-fixed effect	NO	NO	YES	YES
province-fixed effect	NO	NO	NO	YES
N	310	310	310	310
R^2^	0.092	0.295	0.335	0.368

*, **, and *** indicate statistical significance at 10%, 5% and 1% levels, respectively. Robust standard errors are reported in parentheses.

On one hand, the entry of private hospitals enhances the availability of medical resources and expands the variety of services, thereby improving patient access to healthcare, and partially alleviating the issue of “difficulty in accessing healthcare.” Private hospitals typically offer shorter waiting times, and more personalized care, which contributes to improved health outcomes by ensuring timely treatment and medical attention. On the other hand, the entry of private hospitals fosters a competitive environment that incentivizes public hospitals to innovate, improve healthcare delivery, and upgrade their facilities. This competition drives higher standards of care across the healthcare system, thereby benefiting the overall health of the population.

In this study, certain control variables, such as population aging, Population density, health insurance coverage rate, and the development of tertiary hospitals, do not exhibit significant impacts on the health level. The coefficient of educational attainment is significantly negative, indicating that regions with a higher level of educational attainment tend to have better health outcomes. This can be attributed to the fact that higher educational attainment often correlates with healthier lifestyles and improved access to healthcare services. Additionally, the coefficient of the economic development level is significantly negative, suggesting that a higher level of economic development is associated with improved health level. This relationship can be explained by the fact that economic development typically results in better infrastructure, improved healthcare access, and higher incomes, enabling individuals to afford better health services.

### 4.2 Endogeneity test

Omitted variables and measurement errors may introduce endogeneity issues in the model. To mitigate the bias arising from these endogeneity issues, and following the approach of Groves (41), Lv and Zhao (42) for identifying instrumental variables, this study uses the hospital competition from the previous period $L.Comp_{\mathrm{it}}$ as an instrumental variable for the current period. Additionally, the two-stage least squares (2SLS) method is employed to examine the relationship between relaxing entry regulation for private hospitals and health level. The results are presented in Table 4.

**Table 4:** Regression results of IV-2SLS.

Variables	First-stage regression results	Two-stage regression results
	Comp	Health
L. Comp	0.868^***^ (0.034)	
Comp		−1.029^***^ (0.345)
aging	−0.091 (0.111)	4.320 (2.686)
den	0.002 (0.001)	0.030 (0.075)
edu	−0.003 (0.080)	−3.142^**^ (1.332)
insur	0.008 (0.066)	0.252 (1.850)
pgdp	−0.007 (0.033)	−1.499^***^ (0.409)
terh	0.305^**^ (0.123)	2.221 (1.636)
Cons	0.094 (0.409)	18.302^***^ (4.961)
time-fixed effect	YES	YES
province-fixed effect	YES	YES
First-stage F-statistics	648.065	
N	279	279
R^2^	0.942	0.891

*, **, and *** indicate statistical significance at 10%, 5% and 1% levels, respectively. Robust standard errors are reported in parentheses.

Table 4 presents that controlling for all control variables and incorporating double fixed effects, the IV-2SLS estimation reveals that the impact of hospital competition on maternal mortality is significantly negative at the 1% significance level, with an estimated coefficient of −1.029. This suggests that relaxing entry regulation for private hospitals has a positive impact on health level. In comparison to the baseline regression, the effect of relaxing entry regulation for private hospitals on health level is strengthened after addressing the endogeneity issue. An F-statistic of 648.065 in the first stage indicates that the selected instrumental variables are strongly correlated with the endogenous explanatory variables, confirming the absence of weak instrumental variable issues. The results of the instrumental variables regression confirm the robustness of this study’s findings, even after addressing the endogeneity issue.

### 4.3 Robustness test

#### 4.3.1 Replacement of explained variables

To confirm the robustness of the baseline regression results, this study conducts a robustness test by substituting the explained variables. The explained variables *Health* is substituted with the urban maternal mortality rate (*urb_Health*) and the rural maternal mortality rate (*rur_Health*), respectively. Logarithmic transformations are applied. The regression results are presented in Table 5.

**Table 5:** Robustness test (part 1).

Variables	Replacing the explained variable					
	*urb_Health*			*rur_Health*		
	(1)	(2)	(3)	(4)	(5)	(6)
Comp	−1.548^**^ (0.649)	−1.448^**^ (0.665)	−1.572^**^ (0.702)	−1.326^**^ (0.640)	−1.235^**^ (0.595)	−1.143^*^ (0.629)
aging	−0.791 (1.829)	−2.740 (1.831)	3.999 (3.481)	2.860 (3.268)	−3.136 (3.288)	1.690 (3.462)
den	−0.069 (0.084)	−0.092 (0.083)	−0.039 (0.119)	0.061 (0.090)	−0.054 (0.080)	0.065 (0.112)
edu	1.770^*^ (0.904)	0.593 (1.011)	−2.691 (1.746)	2.823 (1.827)	1.475 (1.999)	1.214 (2.370)
insur	−1.139^**^ (0.509)	0.211 (1.033)	0.160 (2.395)	0.676 (1.172)	2.701^*^ (1.573)	2.049 (2.346)
pgdp	−0.754^***^ (0.110)	−1.110^***^ (0.235)	−1.258^**^ (0.509)	−0.641^***^ (0.188)	−1.531^***^ (0.315)	−2.193^***^ (0.583)
terh	−0.574 (2.037)	−0.823 (2.055)	3.501 (2.891)	−5.075^**^ (2.367)	−5.569^**^ (2.199)	−4.297 (3.156)
Cons	11.930^***^ (1.340)	15.860^***^ (2.421)	16.440^**^ (6.103)	9.181^***^ (1.892)	19.624^***^ (3.416)	24.867^***^ (6.132)
time-fixed effect	NO	YES	YES	NO	YES	YES
province-fixed effect	NO	NO	YES	NO	NO	YES
N	307	307	307	301	301	301
R^2^	0.251	0.286	0.319	0.205	0.260	0.284

*, **, and *** indicate statistical significance at 10%, 5% and 1% levels, respectively. Robust standard errors are reported in parentheses.In columns (1) to (3), the sample size is 307, accounting for 3 years of missing data from Tibet. In columns (4) to (6), the sample size is 301 due to 9 years of missing rural health data from Shanghai.

The regression results in columns (1) to (6) of Table 5 reveal that the estimated coefficients of the core explanatory variables are consistently and significantly negative, regardless of the inclusion of province and time-fixed effects or the focus on urban or rural residents. In other words, the entry of private hospitals into the market improves the population’s health level, indicating that the results of the baseline regression are robust. Furthermore, the magnitude of the coefficients of the core explanatory variables in columns (3) and (6) of Table 5, relative to the baseline regression, suggests that the effect of relaxing entry regulation for private hospitals on the health level of urban residents is more pronounced. This may be attributed to urban private hospitals being more advanced and larger than those in rural areas, which results in a stronger health-promoting effect.

The result provides significant policy implications, highlighting the persistent healthcare service disparities between urban and rural residents in China, with rural healthcare institutions significantly lagging behind urban ones in both quantity and quality. The development of private hospitals may offer a novel development approach by incentivizing social capital to invest in constructing diverse healthcare institutions in grassroots and rural areas. This strategy seeks to address deficiencies in grassroots medical and healthcare facilities, allowing a broader grassroots population to gain access to improved local healthcare services.

#### 4.3.2 Substitution of econometric model

Drawing on previous studies (23, 43), and acknowledging the temporal persistence in health levels, this study employs a dynamic panel model as the econometric approach to conduct a robustness test. The model is specified in Equation 4:


(4) $$\mathrm{Healt}h_{\mathrm{it}}=\beta_{0}+\beta_{1}L.\mathrm{Healt}h_{\mathrm{it}}+\beta_{2}\mathrm{qcom}p_{\mathrm{it}}+\beta_{c}\mathrm{Contro}l_{\mathrm{it}}+\mu_{i}+\varepsilon_{\mathrm{it}}$$

Where $L.\mathrm{Healt}h_{\mathrm{it}}$ denotes the lag period of the explained variable, and other variables retain the same meaning as defined in the baseline regression model. In this study, the model is primarily estimated using the one-step system GMM approach, with the results of one-step differential GMM also presented to verify robustness. The test results for the serial correlation of disturbance terms are provided by the *p*-values of AR (1) and AR (2). The over-identification test of instrumental variables is conducted by the *p*-value of the Sragan test. Referring to Arellano and Bond (44), the *p*-value for the Sargan test reported here corresponds two-stage estimation. The regression results are presented in columns (1) and (2) of Table 6.

The regression results of both the system GMM and differential GMM models, as presented in Table 6, indicate that relaxing entry regulation for private hospitals has a significantly positive effect on health level. The empirical findings of this study remain valid after when the estimation method is changed, confirming the robustness of the baseline regression results.

**Table 6:** Robustness test (part 2).

Variables	Substitution of econometric model		Modified sample
	One-step system GMM	One-step differential GMM	
	(1)	(2)	(3)
L. Health	0.353^***^ (0.121)	0.055 (0.094)	
Comp	−2.088^**^ (0.993)	−2.887^**^ (1.460)	−0.857* (0.445)
aging	−3.687 (4.467)	1.849 (2.371)	4.330 (2.576)
den	0.071 (0.178)	0.003 (0.108)	−0.002 (0.053)
edu	−1.057 (1.624)	−1.079 (1.402)	−3.056* (1.586)
insur	−2.316^***^ (0.790)	−5.107 (3.582)	0.451 (2.147)
pgdp	0.077 (0.316)	−0.144 (0.241)	−1.221*** (0.414)
terh	5.706 (3.731)	2.394 (4.334)	1.822 (1.680)
Cons	1.683 (3.708)	6.126^**^ (2.617)	15.565*** (4.592)
AR (1)	0.001	0.005	
AR (2)	0.381	0.609	
Sargan	0.999	0.815	
N	279	248	310
R^2^			0.371

*, **, and *** indicate statistical significance at 10%, 5% and 1% levels, respectively. Robust standard errors are reported in parentheses. L. denotes the lag of one period for the variable.

#### 4.3.3 Data winsorization analysis

To mitigate the influence of outliers on the regression results, this study applies a 1% winsorization treatment to the explained and core explanatory variables. The results are presented in column (3) of Table 6. The coefficient of the core explanatory variable remains significantly negative. These results are consistent with those of the baseline regression, further confirming that the findings are robust even when using a modified sample.

### 4.4 Heterogeneity analysis

#### 4.4.1 Regional heterogeneity

To comprehensively examine the health effect of relaxing entry regulation for private hospitals, this study conducts a detailed heterogeneity analysis based on regional disparity, healthcare resource endowment, and the degree of quality competition. This study divides Mainland China into two geographic regions: the Central-Western region and the Eastern region, according to the regional classification standards outlined in the China Healthcare Statistical Yearbook. The results detailed in columns (1) and (2) of Table 7 suggest that relaxing entry regulation for private hospitals has a significant positive impact on health level in the Central-Western region, whereas the coefficient for the Eastern region is statistically insignificant.

**Table 7:** Results of the heterogeneity analysis.

Variables	(1)	(2)	(3)	(4)	(5)	(6)
	Central-Western region	Eastern region	Low-mre	High-mre	High-quality competition	Low-quality competition
Comp	−0.526^*^ (0.270)	−3.657 (2.497)	−0.459 (0.460)	−2.198^*^ (1.205)	−1.009^***^ (0.238)	−1.334 (1.232)
aging	5.435^**^ (1.946)	0.284 (5.281)	2.691 (2.428)	1.685 (4.111)	4.831^**^ (1.995)	3.834 (4.053)
den	0.026 (0.058)	−0.882 (0.603)	−0.018 (0.103)	−0.027 (0.148)	0.031 (0.064)	−0.138 (0.129)
edu	−1.743^*^ (0.931)	−5.611 (3.366)	0.477 (1.453)	−6.792^*^ (3.218)	−1.042 (1.301)	−4.009 (2.449)
insur	−1.439 (1.841)	−2.123 (1.656)	−0.828 (1.616)	0.699 (2.511)	1.755 (2.149)	−0.637 (1.752)
pgdp	−1.056^**^ (0.438)	−0.545 (1.696)	−1.702^***^ (0.381)	−0.257 (1.414)	−1.116^**^ (0.425)	−0.901 (0.964)
terh	−0.399 (1.752)	5.374 (3.589)	−1.374 (2.970)	5.053 (3.388)	0.105 (1.485)	2.433 (2.985)
Cons	13.725^***^ (4.422)	18.313 (19.905)	20.512^***^ (3.744)	6.785 (15.299)	14.066^***^ (4.246)	13.910 (9.829)
Time-fixed effect	YES	YES	YES	YES	YES	YES
Province-fixed effect	YES	YES	YES	YES	YES	YES
N	200	110	160	150	150	160
R^2^	0.687	0.248	0.660	0.332	0.710	0.236

*, **, and *** indicate statistical significance at 10%, 5% and 1% levels, respectively. Robust standard errors are reported in parentheses.

A potential explanation for this disparity is that, compared to the Eastern region, the Central-Western region suffers from a relative scarcity of healthcare resources, with limited access to high-quality healthcare resources. The introduction of private hospitals into these markets not only substantially increases the supply of healthcare services but also improves residents’ access to medical care, thereby significantly improving health level in the Central-Western region.

#### 4.4.2 Medical resource endowment heterogeneity

Practicing (assistant) physicians represent a crucial component of medical human resources and have a substantial impact on patients’ medical treatment choices. Based on the number of practicing (assistant) physicians per thousand population in 2019 across 31 provinces, this study classifies these provinces into two groups: regions with high medical resource endowment (*High-mre*) and those with low medical resource endowment (*Low-mre*). The results are shown in columns (3) and (4) of Table 7, which reveal that the effects of relaxing entry regulation for private hospitals on health level vary across regions with different healthcare resource endowment.

In regions with high healthcare resource endowment, relaxing entry regulation for private hospitals significantly enhances health level. Conversely, the estimated coefficient for regions with low healthcare resource endowment is not statistically significant. This is because regions with high healthcare resources typically have a higher concentration of private hospitals, which achieve a certain scale through the number of institutions and the volume of services they offer. Consequently, the entry of private hospitals into these markets exerts a significant impact on health outcomes. In contrast, regions with low healthcare resources have relatively fewer private hospitals, and their small scale leads to less pronounced effects on health improvement. The above findings suggest that the imbalance allocation of healthcare resources across different regions in China undermines the fairness of access to medical services, thereby contributing to disparities in health outcomes. Promoting a more equitable distribution and optimizing the structure of healthcare resources should remain a priority for ongoing healthcare system reforms.

#### 4.4.3 Quality competition heterogeneity

To explore whether relaxing entry regulation for private hospitals has heterogeneous effects on health level under varying degrees of quality competition, this study uses the mean proportion of tertiary hospitals in 2019 as the grouping criterion. Regions with a proportion lower than the mean represent a relatively low share of tertiary hospitals, where private hospitals are more competitive and the degree of quality competition is higher (*High-quality competition*). Conversely, regions with a proportion higher than the mean suggest a larger scale of public tertiary hospitals, where the quality competition between public and private hospitals is relatively low (*Low-quality competition*). Regression analysis was performed based on these groupings, and the results are shown in columns (5) and (6) of Table 7.

Column (5) of Table 7 illustrates that in regions with a low proportion of tertiary hospitals (*High-quality competition*), relaxing entry regulation for private hospitals significantly improve the health level. In these regions, the entry of private hospitals into the market increases the supply of medical services, transforming potential demand into actual demand, thereby directly leads to health-promoting effects. Additionally, private hospitals entering the market engage in a degree of quality competition with public hospitals, which may indirectly improve residents’ health by enhancing the quality of healthcare services.

Column (6) of Table 7 reveals that in regions with a high proportion of tertiary hospitals (*Low-quality competition*), relaxing entry regulation increases the number of private hospitals. However, due to their relatively small scale, private hospitals face challenges in effectively competing on quality with large public tertiary hospitals that possess significant market power. Consequently, the health-promoting effect of private hospitals is limited, and relaxing entry regulation for private hospitals does not result in significant improvement in health outcomes in these regions.

## 5 Analysis of the nonlinear characteristics

### 5.1 Threshold effect test

This study uses variables including population aging, population density, educational attainment, health insurance coverage rate, economic development level, and the proportion of tertiary hospitals for threshold measurement. In the panel threshold model, determining the existence and number of thresholds is a critical step. This study employs the Bootstrap resampling method with 300 iterations, beginning with a test for up to three thresholds.

**Figure 2:**
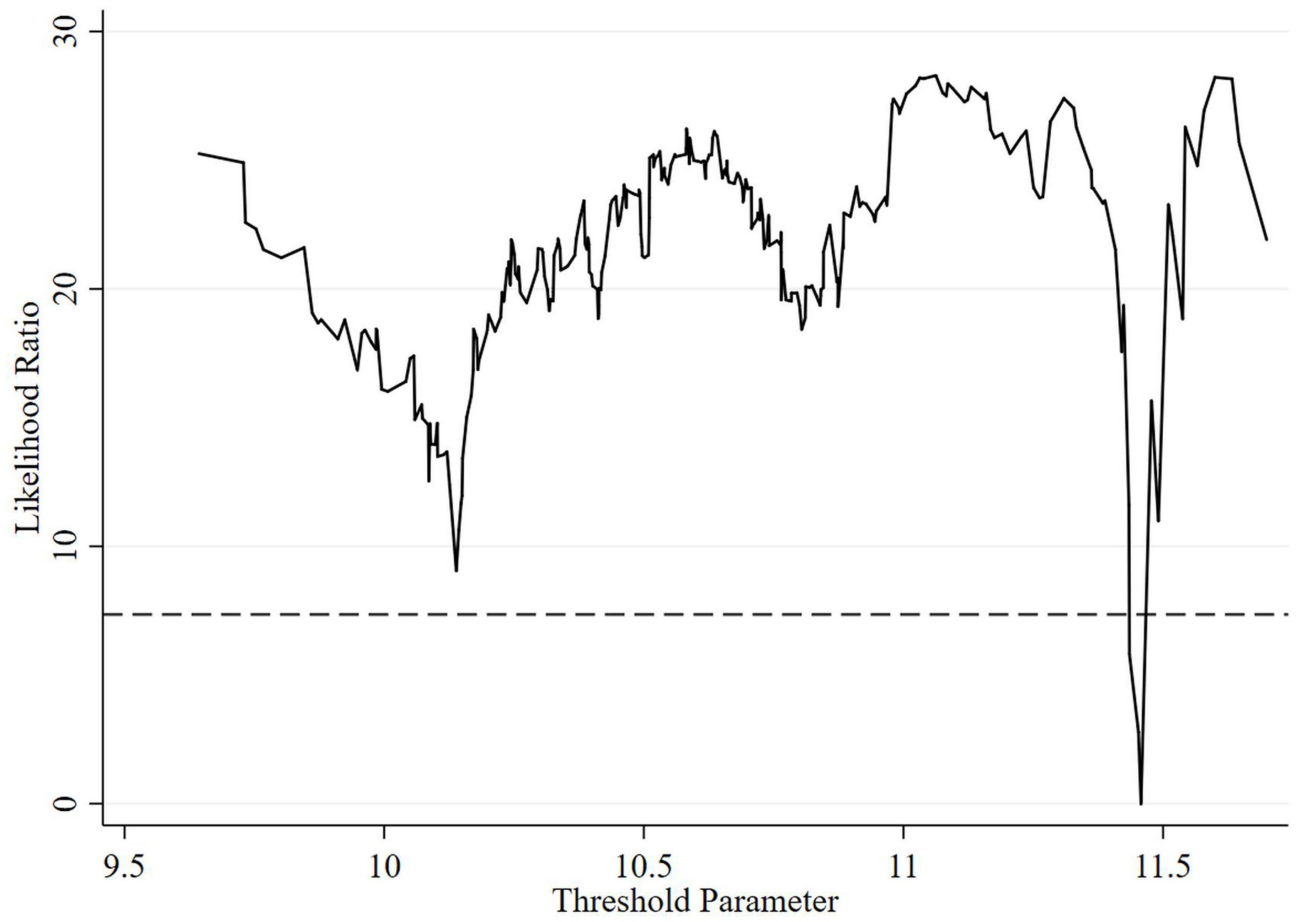
Plot of the LR statistics test for the threshold effect of economic development level.

**Figure 3:**
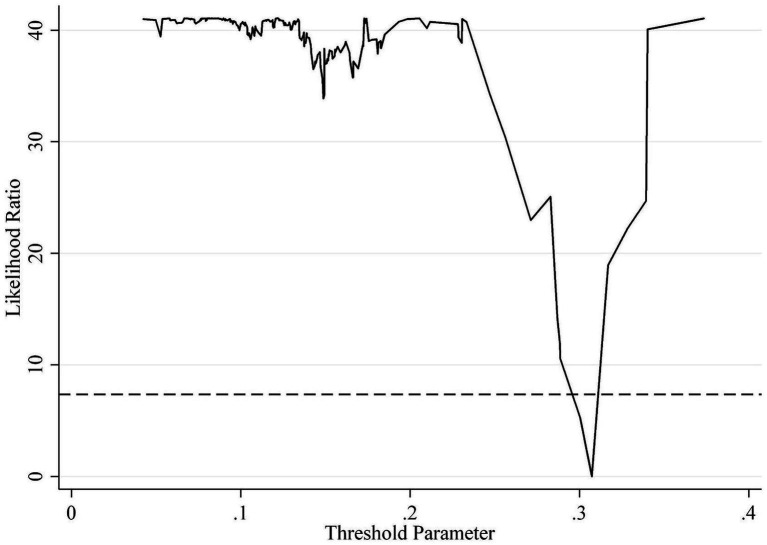
Plot of the LR statistics test for the threshold effect of educational attainment.

As shown in Table 8, the threshold effect test identifies a threshold value of 11.4575 for economic development level and 0.3073 for educational attainment, whereas the other variables fail to pass the threshold effect test. The LR statistics test charts more clearly display the number of thresholds and the magnitude of their values for economic development level and educational attainment. Accordingly, this study conducts a graphical analysis of the LR statistics for economic development level and educational attainment, as illustrated in Figures 2, 3, respectively.

**Table 8:** Threshold effect test and threshold value estimation.

Threshold variables	Threshold value	*F*-value
pgdp_it_	γ_1_ = 11.458	28.980** (0.013)
edu_it_	γ_2_ = 0.307	42.040* (0.043)

*, **, and *** indicate statistical significance at 10%, 5% and 1% levels, respectively. *p*-values are shown in parentheses, and Bootstrap resampling was conducted 300 times.

### 5.2 Analysis of the nonlinear characteristics

To comprehensively investigate how economic development level and educational attainment influence the causal relationship between relaxing entry regulation for private hospitals and health level, this study employs a panel threshold model to analyze the nonlinear characteristics. The results are presented in Table 9.

**Table 9:** Analysis of threshold effect.

Variables	(1)	(2)
Comp_it_ (pgdp_it_ ≤ 11.4575)	−1.187^***^ (0.360)	
Comp_it_ (pgdp_it_>11.4575)	−0.084 (0.698)	
Comp_it_ (edu_it_ ≤ 0.3073)		−0.752 (0.445)
Comp_it_ (edu_it_>0.3073)		−3.188^***^ (0.504)
aging	−0.758 (2.345)	6.969^***^ (1.671)
den	0.098 (0.081)	−0.002 (0.065)
edu	−4.351^**^ (1.798)	
insur	−2.739^**^ (1.201)	−0.801 (2.127)
pgdp		−0.854^***^ (0.105)
terh	0.645 (1.810)	0.587 (1.621)
Cons	3.402^***^ (0.785)	11.518^***^ (1.228)
N	310	310
R^2^	0.310	0.393

*, **, and *** indicate statistical significance at 10%, 5% and 1% levels, respectively. Robust standard errors are reported in parentheses.Bootstrap resampling was repeated 300 times.

The regression results in Table 9 reveal that the health effect of relaxing entry regulation for private hospitals exhibits significant interval characteristics, rather than a simple linear relationship, depending the levels of economic development and educational attainment. The regression results in column (1) of Table 9 indicate that relaxing entry regulation for private hospitals significantly improves health level when the economic development level is below the threshold value of 11.4575. However, once the economic development level surpasses the threshold, the health-promoting effect of relaxing entry regulation for private hospitals becomes insignificant. These findings suggest that the impact of relaxing entry regulation for private hospitals on improving public health diminishes as economic development levels increase. The above conclusions indicate that achieving the goal of a Healthy China requires a comprehensive implementation of various policies to enhance the effectiveness of health promotion.

Column (2) of Table 9 reveals that when educational attainment is below the threshold of 0.3073, the health-promoting effect of relaxing entry regulation for private hospitals is insignificant. However, when the educational attainment exceeds this threshold, relaxing entry regulation for private hospitals significantly enhances residents’ health levels. One possible explanation for this is that as education levels increase, residents’ focus on and knowledge of health also improve, thereby significantly bolstering the health-promoting effect of relaxing entry regulation for private hospitals. The above analysis demonstrates that once educational attainment reaches a certain threshold, it becomes more conducive to the health-promoting effect of private hospitals. These results support Hypothesis 2.

Based on the relationship between economic development levels and educational attainment relative to the threshold values, this study categorizes provinces in China into three typical region types.

1. The first category comprises the “two high” regions, characterized by both economic development level and educational attainment generally exceeding the threshold values, as exemplified by Beijing and Shanghai. In these regions, the health effects of relaxing entry regulation for private hospitals are relatively high, necessitating a focus on promoting high-quality development of private hospitals and maintaining their role in enhancing health. As economic development reaches a certain level, the health-promoting effect of relaxing entry regulation for private hospitals become relatively limited. Consequently, health policies in these regions should emphasize improving the quality of patient care, through measures such as reforming public hospitals and facilitating the redistribution of high-quality healthcare resources, thereby enhancing health promotion more effectively.
2. The second category comprises the “two lows” regions, characterized by both economic development level and educational attainment typically falling below the threshold value, such as Anhui, Fujian, Gansu, Guangxi, Guizhou, Hebei, Henan, and Jiangxi. The health effect of relaxing entry regulation for private hospitals in these regions still holds considerable potential for enhancement. Alongside improving economic development level and educational attainment, it is also necessary to enhance support for private hospital development. This requires the formulation and implementation of policies that promote and regulate private hospital growth, thereby increasing healthcare service supply, meeting patient demand, and ultimately improving public health.
3. The third category comprises the “one high and one low” regions, where economic development level exceeds the threshold value, but educational attainment remains below the threshold value, as observed in provinces such as Tianjin, Jiangsu, Zhejiang. In these regions, the impact of economic development on the health effects of relaxing entry regulation for private hospitals is relatively limited, whereas improvements in educational attainment have a more pronounced influence on the health effects of relaxing entry regulation for private hospitals. Consequently, while continuing to advance the development of private hospitals, it is essential for these regions to prioritize strategies for further improving educational levels.

## 6 Conclusion and discussion

### 6.1 Conclusion

Grounded in the policy context and specific characteristics of relaxing entry regulation for private hospitals, this study utilizes Chinese empirical data to comprehensively assess and systematically analyze the health effects. The study’s conclusions encompass the following four key aspects. (1) The relaxation of entry regulation for private hospitals significantly improves residents’ health level, a finding empirically validated across both urban and rural populations. (2) Heterogeneity analysis indicates that the health-promoting effect of relaxing entry regulation for private hospitals are particularly pronounced in Central-Western region, regions with high healthcare resource endowment, and regions with high-quality competition. (3) The threshold effect test reveals that the health effect of relaxing entry regulation for private hospitals exhibit nonlinear characteristics. Health benefits diminish when economic development level exceeds a specific threshold, whereas become more pronounced when educational attainment reaches a certain benchmark. (4) According to the relationship between economic development level, educational attainment, and threshold values across provinces, China’s provinces can be classified into three distinct regions to facilitate the formulation of targeted policies.

### 6.2 Policy implications

Based on the research findings, this study proposes three key policy insights that are crucial for establishing a fair competition mechanism in the healthcare services market, enhancing the diversity of medical services supply, and improving residents’ health.

1. Completely eliminate institutional barriers to private hospitals market entry and establish a fair competition mechanism in the healthcare market. The empirical research of this study demonstrates that relaxing entry regulation for private hospitals significantly promotes health level. Although China has issued several policy documents in recent years to relax market entry for private hospitals and remove policy barriers, the practical implementation of these policies has faced numerous obstacles and failed to achieve the expected results. Although the number of private hospitals has surpassed that of public hospitals, their development scale remains small and their market competitiveness weak. Public hospitals in China continue to dominate position in healthcare resource allocation, asset scale, and market share. Currently, China continues to operate under an administrative monopoly dominated by public hospitals. Therefore, it is imperative to accelerate the implementation of measures to promote private hospital development. This includes creating a systematic environment conducive to private investment, reducing administrative entry barriers, and ensuring private hospitals receive equal treatment with public hospitals in all respects, thereby fostering a diversified and competitive medical service system.
2. Tailor development strategies for private hospitals based on the specific developmental characteristics of various regions. Based on the results of heterogeneity analysis, it is essential to strengthen political and financial support for the development of private hospitals in Central-Western region and regions with scarce healthcare resources. Relaxing entry regulation increases the number of private hospitals, however, their development scale remains limited, and they primarily fulfill their health promotion function in regions with fewer tertiary hospitals. Consequently, encouraging social capital to establish high-quality private medical institutions at the grassroots level can enhance the supply of primary healthcare services, create a complementary market dynamic with public hospitals, and fully leverage the health-promoting potential of private hospitals at the grassroots level. The threshold model indicates that the health effect of relaxing entry regulation for private hospitals is influenced by both economic development level and educational attainment. Under the overarching policy of promoting private hospital development, regional governments should formulate customized implementation strategies tailored to their specific economic and educational contexts to maximize the health-promoting effect of private hospitals.
3. Strengthen the quality regulation of healthcare services in private hospitals to promote their high-quality and large-scale development. Simply relaxing entry regulation for private hospitals is insufficient to fully unlock their potential, requiring a series of supportive measures to effectively enhance their market competitiveness through integrated policies. This study’s findings indicate that enhancing quality competition is essential for fully leveraging private hospitals’ potential and promoting the joint and coordinated development of public and private hospitals. Furthermore, in a healthcare market characterized by asymmetric information, healthcare resources partially reflect the quality of medical care. Reducing the disparities in resource allocation between private and public hospitals, while fostering the development of major private hospitals, is essential for enabling their transition to high-quality and large-scale operations. Although private hospital development contributes to improving public health, it is crucial for the Chinese government to prioritize its regulatory role in the healthcare system. At the same time, it is imperative to strengthen quality regulation for private hospitals to ensure the quality of their healthcare services, thereby enhancing their role in health promotion.

### 6.3 Limitations and future research

Although this study provides valuable insights into the effects of relaxing entry regulation for private hospitals on health level in China, it has certain limitations. First, this study mainly analyses the effect of relaxing entry regulation for private hospitals on health based on panel data from 31 provinces in mainland China. Future research should focus on exploring the potential mechanisms and specific pathways through which the development of private hospitals improves health level, offering new insights to refine the policies for private hospital development.

Second, this study primarily employs the fixed effects model and panel threshold model to reveal the relationship between the relaxation of entry regulation for private hospitals and health level, as well as its nonlinear characteristics. While it provides a valuable starting point, future research could diversify research methods by introducing case studies of medical institutions and conducting patient questionnaires. Such methods could offer a more comprehensive understanding of the effects of relaxing entry regulation for private hospitals.

Third, while this study primarily focuses on the health effect of relaxing entry regulation for private hospitals, considering the fact that competition in China’s medical market is dominated by government policies, future research could further explore the potential of private hospitals to improve medical service quality and control medical costs in an evolving medical policy environment. As the role of the government in the medical system continues to evolve, the development of private hospitals may encounter new opportunities and challenges.

## Data Availability

The original contributions presented in the study are included in the article/supplementary material, further inquiries can be directed to the corresponding author.
